# Considerations on Structural Vaccinology and Epitope Screening of Calcium-Dependent Protein Kinases 8 as a Potential Vaccine Target Against *Toxoplasma gondii*

**DOI:** 10.1155/ipid/4426082

**Published:** 2025-09-09

**Authors:** Amirhossein Abedi, Ali Dalir Ghaffari

**Affiliations:** ^1^Student Research Committee, Faculty of Medicine, Shahed University, Tehran, Iran; ^2^Department of Parasitology and Mycology, Faculty of Medicine, Shahed University, Tehran, Iran

**Keywords:** bioinformatics, CDPK8, in silico, *Toxoplasma gondii*

## Abstract

**Introduction:** *Toxoplasma gondii* (*T. gondii*) is a widely prevalent parasite from the phylum apicomplexan and is the causative agent of toxoplasmosis, which affects almost all warm-blooded animals, including humans. Presently, conventional treatments for toxoplasmosis have limited effectiveness against the cystic forms of the parasite. Thus, developing an efficient and safe vaccine for control and prevention of toxoplasmosis is crucial. Calcium-dependent protein kinases (CDPKs) are essential in governing crucial biological processes like anchoring to host cell, cellular infiltration, dynamic locomotion, and escape mechanisms. Because there are no reports on immunization with CDPK8 to date, this study evaluated the fundamental biochemical traits and immunogenic epitopes of the CDPK8 protein through diverse bioinformatics tools.

**Materials and Methods:** We examined the physicochemical attributes, antigenicity, potential B- and T-cell epitopes, tertiary and secondary structures, transmembrane domains, subcellular localization, allergenicity, and other characteristics of the CDPK8 protein.

**Results:** CDPK8 exhibited notable surface accessibility, flexibility, antigenicity, and hydrophilicity indices. Epitope prediction results from diverse bioinformatics databases revealed multiple premiums T-cell and B-cell within the CDPK8 protein shows its viability as an essential component in a *T. gondii* vaccine formulation. Our findings suggest that to minimize the risk of errors and failures in the laboratory, utilizing in silico software for predicting the functional and structural properties of the CDPK8 protein could be a crucial and essential step in preventing cost wastage.

**Conclusion:** To confirm the immunogenicity of the anticipated sequences, validation in an appropriate mouse model using various bioinformatics tools is recommended. Therefore, it is highly recommended that this protein be evaluated in silico and biological platforms settings to characterize its structural and immunological roles for potential prophylactic agent.

## 1. Introduction

*Toxoplasma gondii* (*T.* gondii) is a widely prevalent parasite from the phylum apicomplexan and is the causative agent of toxoplasmosis, which affects almost all warm-blooded animals, including humans, while domestic and wild felids serve as the definitive host [1]. Globally, *T. gondii* has affected around one-third of the humans. Oocysts serve as the possible infective type in the parasite's life cycle. The dissemination of nonsporulated oocyst contamination can occur through the feces of felids, which serve as the primary hosts, into the surroundings [2]. *T. gondii* has three infectious stages including bradyzoites (tissue cyst form), sporozoites (in oocysts), and tachyzoites (fast multiplying type) [3]. Tachyzoites are active during the acute phase, while bradyzoites are linked to the disease's chronic phase [4]. Following maternal infection, the fetus is at risk of vertical transmission; potentially, women who have come into contact with *Toxoplasma* during pregnancy may experience complications, possibly leading to miscarriage. Importantly, the health risks vary depending on the gestational age and can include issues like developmental delays, microcephaly, hydrocephalus, brain lesions, and hearing impairment [5–8]. Presently, conventional treatments for toxoplasmosis can only inhibit the growth of tachyzoites early in the infection, with limited effectiveness against the cystic forms of the parasite in host tissues [9, 10]. Moreover, administering these medications to pregnant women can result in severe side effects, including potential harm to the developing fetus [11]. Thus, developing an efficient and safe vaccine to manage and prevent toxoplasmosis is crucial, particularly for domestic animals and humans [12]. The only officially recognized vaccine for *Toxoplasma* (TOXOVAX) to date has been specifically designed for congenital diseases in ewes, utilizing the live attenuated S48 strain. Numerous vaccination trials, like protein, DNA, subunit, and various live attenuated vaccines, have been carried out to immunize against toxoplasmosis; however, they have only offered partial protection. Despite exploring different antigens from various components of *T. gondii*, such as rhoptries, micronemes, surface antigens, and dense granules organelles, researchers have not yet discovered a targeted prophylactic treatment for human *T. gondii* infection. Calcium-dependent protein kinases (CDPKs) are linked to calcium signaling, demonstrate a conserved regulatory mechanism, and are essential in governing crucial biological processes like anchoring to host cell, cellular infiltration, dynamic locomotion, and escape mechanisms [13]. A group of CDPKs has garnered significant interest as potential targets for drug development in apicomplexans [14]. While there are no reports on immunization with CDPK8 to date, several studies have shown that CDPK2 [15], CDPK1 [16], CDPK3 [17–19], CDPK5 [20], and CDPK6 [21] vaccination has elicited robust cellular and humoral reactions, prolonging survival in mice. These CDPKs are crucial in apicomplexan parasites and cannot be found in mammalian hosts, making them promising targets for drug development against *T. gondii*-related apicomplexans. Certain selective inhibitors aimed at these enzymes have already been engineered [22]. One of the most crucial steps in vaccine formulation is the prediction of epitopes, as they play a key role in determining the immunogenic potential of antigens. Employing bioinformatics tools and online platforms can assist scientists in forecasting and pinpointing potential B- and T-cell epitopes. Bioinformatics has risen as a prominent interdisciplinary domain that utilizes mathematics, computer science, statistics, biology, physics, medical technologies, and algorithms to scrutinize biological data [23–26]. Consequently, this computational study evaluated the fundamental biochemical traits and immunogenic epitopes of the CDPK8 protein through diverse bioinformatics tools.

## 2. Materials and Methods

### 2.1. Peptide Chain Composition

The National Center for Biotechnology Information (NCBI) public database provided the CDPK8 complete peptide chain composition (https://www.ncbi.nlm.nih.gov/protein/). This study was approved by the Ethical Committee of Shahed University (IR.SHAHED.REC.1402.139). Links to all bioinformatics web servers used in this study are provided in Supporting Table 1.

### 2.2. Prediction of Physicochemical Properties

The Expasy ProtParam forecasted the physicochemical features of CDPK8. It offers insights on aliphatic index (AI), amino acid count, extinction coefficients, protein's pI (isoelectric point), the instability index (II), grand average of hydropathicity (GRAVY), molecular weight (MW), in vivo and in vitro stability half-life, and the count of residues with negative and positive charges [27].

### 2.3. Post-Translational Modification (PTM) Regions of CDPK8

The prediction of PTM sites in the N-glycosylation, proteins, phosphorylation, O-glycosylation, and acetylation was performed respectively using NetNGlyc 1.0 [28], NetOGlyc 4.0 [29], NetPhos 3.1 [30], and GPS-PAIL 2.0 [31] tools. We used our default predicting parameter for the considered tools; however, acetylation and NetNGlyc were exceptions. A basis was found regarding “all forms” and “every Asn residue”.

### 2.4. Subcellular Localization and Transmembrane Domains of CDPK8

The TMHMM 2.0 and PSORT II forecasted the subcellular localization and likely *T. gondii* CDPK8 protein transmembrane domains. Additionally, we examined transmembrane domains using The DeepTMHMM tool [32].

### 2.5. Forecasting Secondary and Tertiary Structures

The Garnier Osguthorpe Robson (GOR) tool anticipated the CDPK8 protein secondary structure [33, 34]. Moreover, the SCRATCH service predicted of disulfide bond [35, 36]. Then, the SWISS-MODEL constructed the protein sequence 3D models using a homology-modeling approach [37].

### 2.6. Optimization and Verification of the Tertiary Structure

The most accurate 3D model was optimized by the GalaxyRefine tool [38], following the CASP10-evaluated refinement approach [39]. It can rehash structure disturbance, and then there is a complete structural relaxation by simulating dynamics [40]. The Ramachandran plot validated protein's 3D configuration by the SWISSMODEL tool [41]. It graphically illustrates energetically favored sites of dihedral angles of the backbone in relation to protein amino acid residues (https://swissmodel.expasy.org/assess/help). Additionally, the refined structure's general quality was approved by ProSA-web. It assigns an overall score to specific structures, with scores that deviate from the anticipated range suggesting possible errors in the predicted protein [42].

### 2.7. Anticipation of Spatial (Discontinuous) and Linear (Continuous) B-Cell Epitopes

The ABCpred tool (threshold: 0.85) with an overlapping filter identified linear B-cell epitopes [43]. It forecasts antigen B-cell epitope(s) by leveraging an artificial neural network (ANN). The BCPREDS, along with SVMTriP, tools predicted continuous B-cell epitopes [44]. These applications function based on distinct criteria: an epitope with 20 amino acids, 75% specificity, and the integration of an overlap filter. Additionally, the BcePred service detected B-cell epitopes, taking into account several physicochemical factors including surface exposure, polarity, flexibility, accessibility, hydrophilicity, turns, and antigenic propensity [45]. The ProtScale tool B performed cell epitope prediction to visually assess epitopes concerning alpha helix, mean flexibility, beta-turn, the percentage of catchable residues, and hydrophobicity [46]. Moreover, hydrophilicity [47], Additionally, the immune epitope database (IEDB) used various tools to predict linear B-cell epitopes with BepiPred [48], evaluate antigenicity [49], to determine how likely a protein is to trigger an immune response, assess surface accessibility [50], to identify exposed regions of the protein that antibodies can target, look at beta-turns [51], which are important for flexible areas of proteins that can be recognized by the immune system, and measure flexibility [52] to find regions of the protein that can change shape and interact more easily with immune receptors.

Conformational B-cell epitopes were predicted by the ElliPro tool from the IEDB. (0.5 min-score; 6 Å max distance). It evaluates epitopes by taking into account factors such as the residual protrusion index, protein structure, and the clustering of neighboring residues [53]. Finally, antigenicity, water solubility and allergenicity, were evaluated using VaxiJen 2.0, AllergenFP 1.0 [54], and PepCalc tools, respectively.

### 2.8. Anticipation of Major Histocompatibility Complex (MHC)-Specific Epitopes, IFN-γ, and IL-4 Inducing Process

The IEDB recommended 2.22 predicted peptides from CDPK8 with an affinity to MHC-I and MHC-II molecules according to the half-maximal inhibitory concentration (IC_50_) value. MHC-I epitopes, each consisting of 10 amino acids, were predicted for H2-Kb, H2-Db, H2-Dd, H2-Kk, H2-Kd, and H2-Ld mouse alleles, while MHC-II epitopes of 15 amino acids were forecasted for HLA-DQ, H-2-I, HLA-DP, and HLA-DR mouse alleles [55]. Afterward, the VaxiJen 2.0, IL4-pred, and IFNepitope tools were used to predict the antigenicity and the potential for IL-4 and IFN-γ induction of the epitopes.

### 2.9. Anticipation of Cytotoxic T-Lymphocyte (CTL) Epitopes

The CTLpred tool was used to analyze and predict the specific CTL epitopes for CDPK8 of *T. gondii* with a reported accuracy of 75.8% and a mixed method. The standard prediction configurations comprised a support vector machine (SVM) value of 0.36 and an ANN value of 0.51 [56]. The collective prediction approach exhibited a precision of 77.6% (https://crdd.osdd.net/raghava/ctlpred/about.html). Additionally, the immunogenicity of the epitope was evaluated using the IEDB MHC Class I immunogenicity prediction tool, which helped determine its potential to stimulate an immune response [55].

### 2.10. Assessment of Allergenic and Antigenic Profiles and Solubility Valuation

The VaxiJen 2.0 and ANTIGENpro assessed protein antigenicity [57]. ANTIGENpro's prediction is primarily based on microarray analysis data and does not rely on alignment or pathogen information. VaxiJen uses a unique, alignment-free method to predict whether proteins are antigenic. This method converts sequences into a specific format that displays various amino acid properties (such as their chemical characteristics) in a numerical and organized way, using auto cross-covariance to capture the key features of the amino acids. The prediction accuracy of this method ranges from 70% to 89% (https://www.ddg-pharmfac.net/vaxijen/VaxiJen/VaxiJen_help.html).

The allergenicity of CDPK8 was assessed using the AllergenFP 1.0 tool, which utilizes a descriptor-associated fingerprint approach with an 88.9% forecast accuracy (https://ddg-pharmfac.net/AllergenFP/). Additionally, the AlgPred tool [54, 58] predicted the CDPK8 protein allergenicity, utilizing six distinct methods. A combination method (IgE epitope + ARPs BLAST + SVMc + MAST) was employed, achieving an 85% accuracy at −0.4.

Additionally, the SOLpro tool was used to predict the solubility of the protein following its overexpression, providing valuable insights into its potential behavior in solution [59]. SOLpro utilizes a two-step SVM architecture considering various presentations of the primary sequence to forecast the solubility of protein in *Escherichia coli* (*E. coli*) when overexpressed (https://scratch.proteomics.ics.uci.edu/explanation.html#SOLpro).

### 2.11. Immune Simulation

The prediction was carried out at three key locations: bone marrow, thymus, and lymph nodes, using the Position-Specific Scoring Matrix (PSSM) and machine learning algorithms [60]. The default settings were configured with a random seed of 12,345, 1050 simulation steps, and a simulation volume of 50. In addition, for assessing the inclusion of CDPK8, the simulation was set up to administer doses at four-week intervals, with time points at 1, 84, and 168, each corresponding to eight simulation steps.

## 3. Results

### 3.1. General Properties of *T. gondii* CDPK8 Gene

The *T. gondii* CDPK8 protein sequence (accession ID: TGME49_ EPT28117.1) comprises 1498 peptide residues, with a MW of 16.142110 kDa and a predicted isoelectric point (pI) of 5.75. The sequence contains 175 positively charged residues (Lys + Arg) and 203 negatively charged residues (Glu + Asp). The sequence contains 22,404 atoms, with an extinction coefficient of 134,935 M^−1^ cm^−1^ when measured in a water medium at 280 nm. CDPK8 is predicted to have a half-life exceeding 20 h in yeast in vivo, 30 h in mammalian reticulocytes in vitro, and more than 10 h in *E. coli* in vivo. The instability calculation classifies the protein with a value of 62.89, indicating its unstable nature. Moreover, the protein's AI and GRAVY values are 73.20 and −0.471, respectively.

### 3.2. Forecasting of PTM Areas of CDPK8

**Figure 1:**
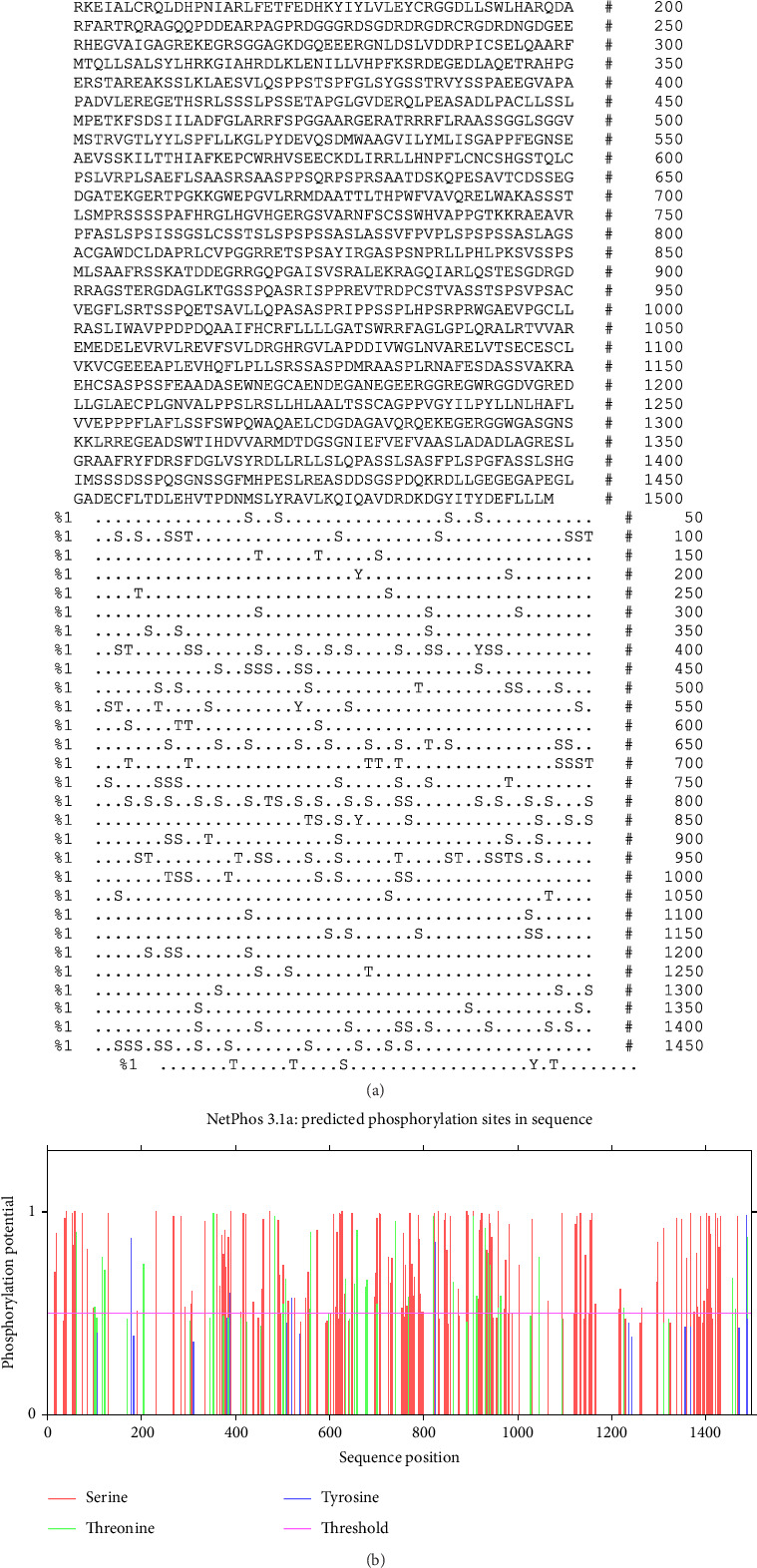
The output from NetPhos server for phosphorylation sites of CDPK8.

**Figure 2:**
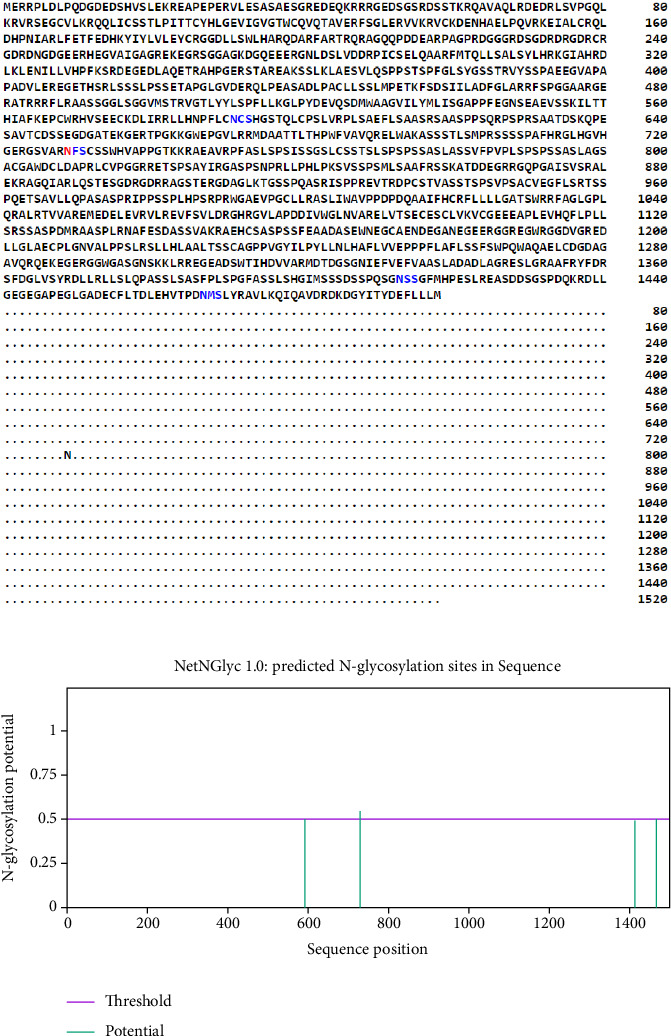
The output from NetNGlyc server for N-glycosylation sites of CDPK8.

The NetPhos 3.1 and GPS-PAIL assessment showed 198 phosphorylation regions (serine 159; threonine 34; and tyrosine 5) and sixteen acylation sites in the sequence, rendering 214 PTM sites (Table 1 and Figure 1). Also, there were 13 N-glycosylation sites (Figure 2) and 132 O-glycosylation regions in the CDPK8 sequence.

### 3.3. Prediction of Subcellular Compartmentalization and Transmembrane Domain

**Figure 3:**
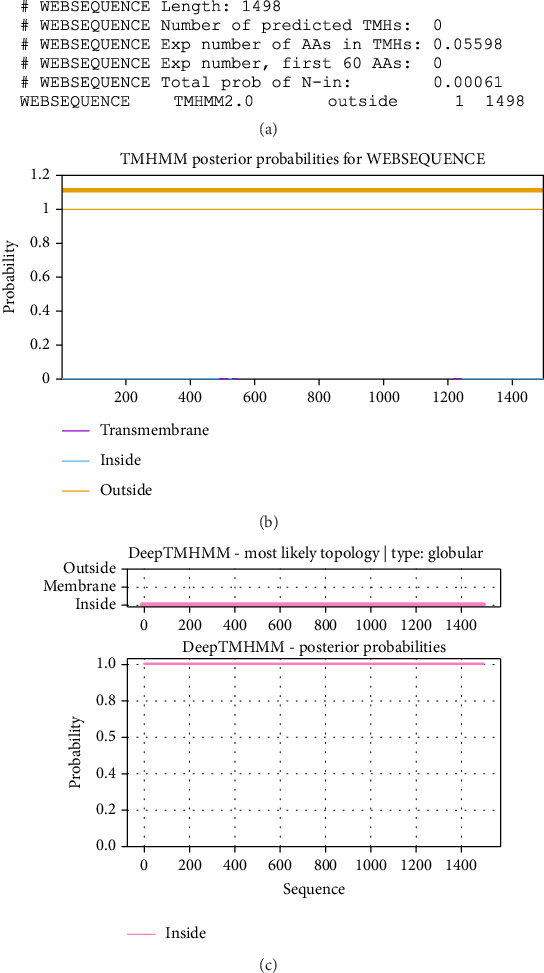
Bioinformatics analysis of the transmembrane domain of CDPK8 sequence. (a) Number of predicted TMHs: the number of predicted transmembrane helices; exp number of AAs in TMHs: the expected number of amino acids in transmembrane helices. If this number is larger than 18, it is very likely to be a transmembrane protein (OR have a signal peptide); exp number—first 60 AAs: the expected number of amino acids in transmembrane helices in the first 60 amino acids of the protein. If this number is more than a few, you should be warned that a predicted transmembrane helix in the N-term could be a signal peptide; total prob. of N-in: the total probability that the N-term is on the cytoplasmic side of the membrane; POSSIBLE N-term signal sequence: a warning that is produced when “Exp number—first 60 AAs” is larger than 10; (b) graphical illustration of transmembrane domain analysis of CDPK8. (c) Analysis of the transmembrane helices of CDPK8.

The TMHMM tool showed no transmembrane domain in the CDPK8 protein. In addition, the results of the DEEPTMHMM tool for analysis of the transmembrane helices are shown in Figure 3. Furthermore, the following subcellular localization findings were obtained: 21.7% cytoplasmic, 65.2% nuclear, 8.7% plasma membrane, and 4.3% cytoskeletal.

### 3.4. Secondary and Tertiary Structure Assessment

**Figure 4:**
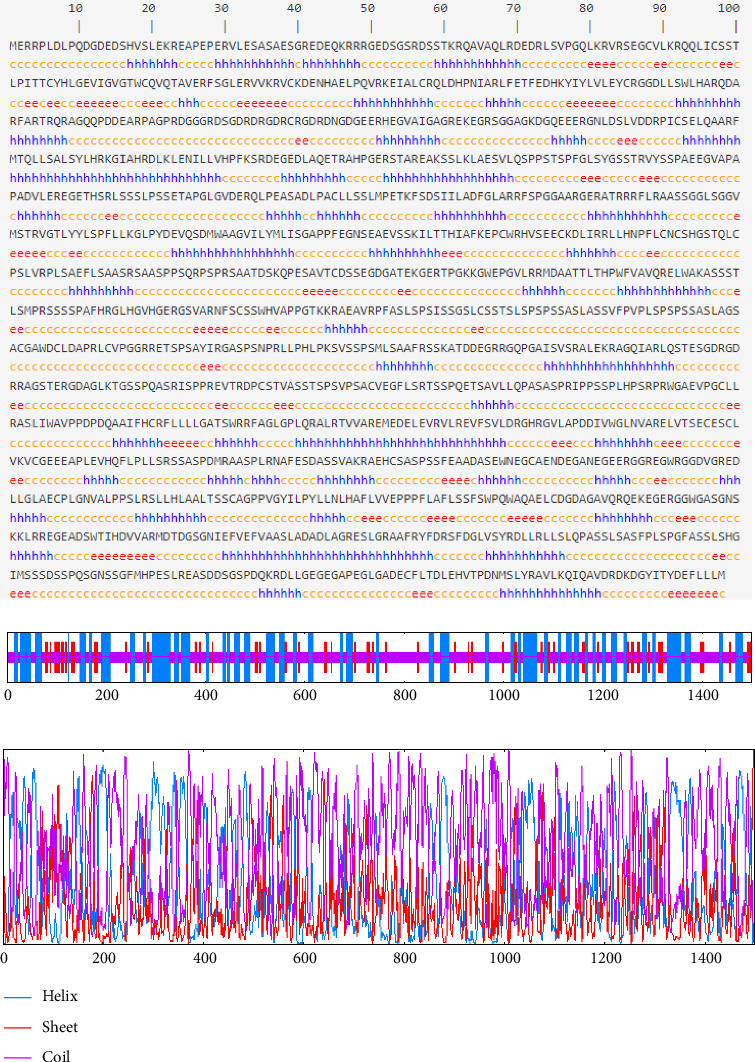
Graphical output of the secondary structure prediction of CDPK8 using GOR IV online servers.

The GOR IV tool reveals 470 (31.38%) alpha helices, 151 (10.08%) extended strands, and 877 (58.54%) random coils (Figure 4). Additionally, the SCRATCH service indicates that our sequence contains a total of 34 cysteines. The cysteines possibly generate the disulfide bond at these positions: 88, 97, 106, 119, 139, 157, 185, 239, 292, 568, 576, 592, 600, 732, 765, 807, 814, 935, 950, 998, 1020, 1096, 1099, 1104, 1153, 1172, 1207, 1231, 1275, and 1455. The top 15 predicted cysteine pairs for disulfide bond formation are ordered by probability in descending order: 139–1455, 935–950, 568–576, 732–765, 88–1275, 97–1231, 239–292, 807–814, 119–1207, 106–1172, 998–1020, 185–1153, 1096–1099, 592–600, and 157–1104 (Table 2).

**Figure 5:**
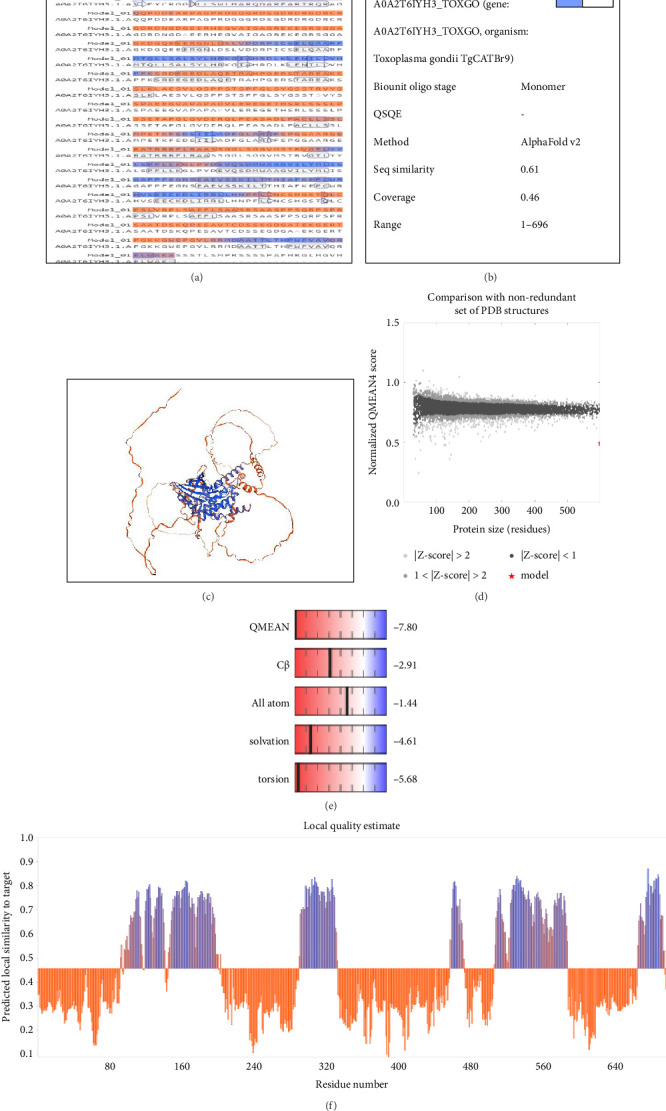
SWISS-MODEL server output. (a) Model-template alignment; (b) sequence identity and coverage; (c) computed three-dimensional model; (d) global quality estimate; (e) comparison with nonredundant set of PDB structures; and (f) local quality estimate.

Nine models were forecasted, and the model characterized by the greatest coverage and 99.14% sequence similarity was the best model.  Figure 5 shows detailed information regarding the SWISS-MODEL evaluation.

### 3.5. Optimization and Verification of the Tertiary Structure

**Figure 6:**
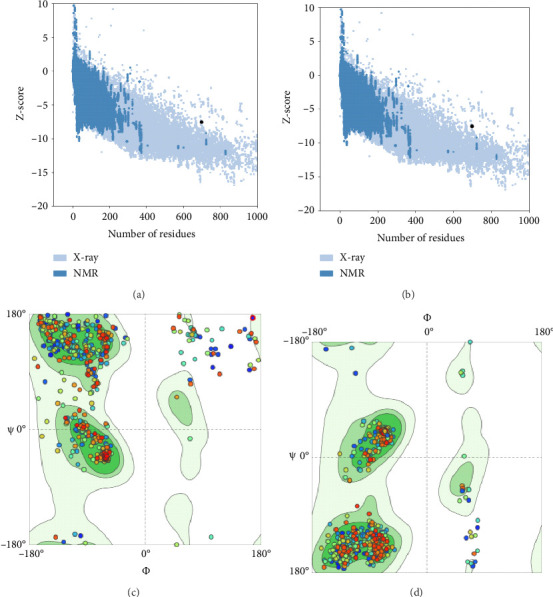
Validation of the 3D structure of CDPK8 protein using Ramachandran plot. (a) The Z-score plot for 3D structure of predicted vaccine before refinement was assessed to be −7.08. (b) The Z-score plot for 3D structure of predicted vaccine after refinement was assessed to be −7.5. The analysis of Ramachandran plot using SWISS-MODEL server in initial model (c), and the model after refinement (d).

According to a comparative analysis of the refinement criteria for different models, the model 1 exhibited the best desirable optimized structure, with GDT-HA (0.8631), poor rotamers (0.3), MolProbity (1.504), RMSD (0.733), clash score (4.8), and Rama favored (96.3) values superior to those of the other models. The ProSA-web and Ramachandran Expasy results showed a Z-score of −7.08 for the initial model, with 82.71% of residues falling within the favored region (Figures 6(a) and 6(c)). After refinement, the 3D structure's Z-score improved to −7.5, with 96.25% of residues in the desirable site (Figures 6(b) and 6(d)).

### 3.6. Shape-Dependent and Continuous B-Cell Epitopes

**Figure 7:**
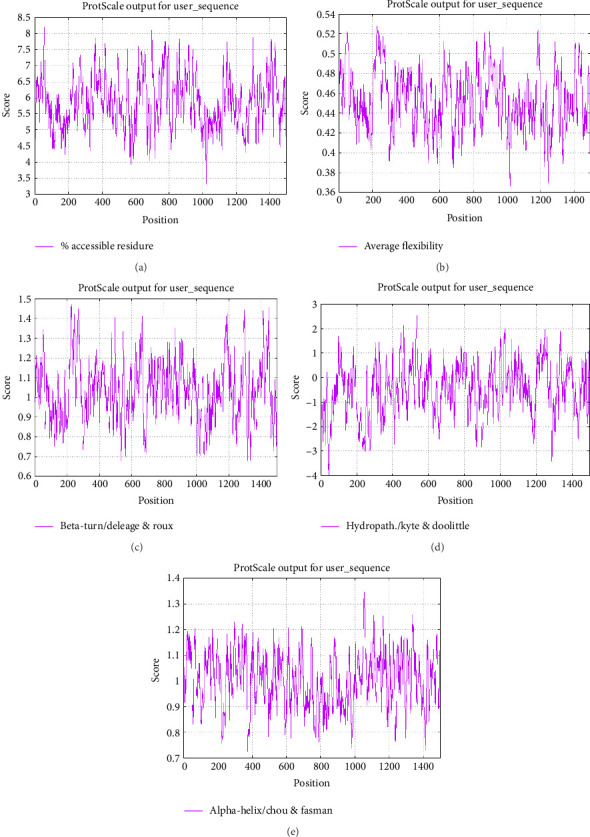
Linear B-cell epitopes of the CDPK8 protein sequence based on (a) percent of accessible residues, (b) average flexibility, (c) beta turn, (d) hydrophobicity, and (e) alpha-helix.

**Figure 8:**
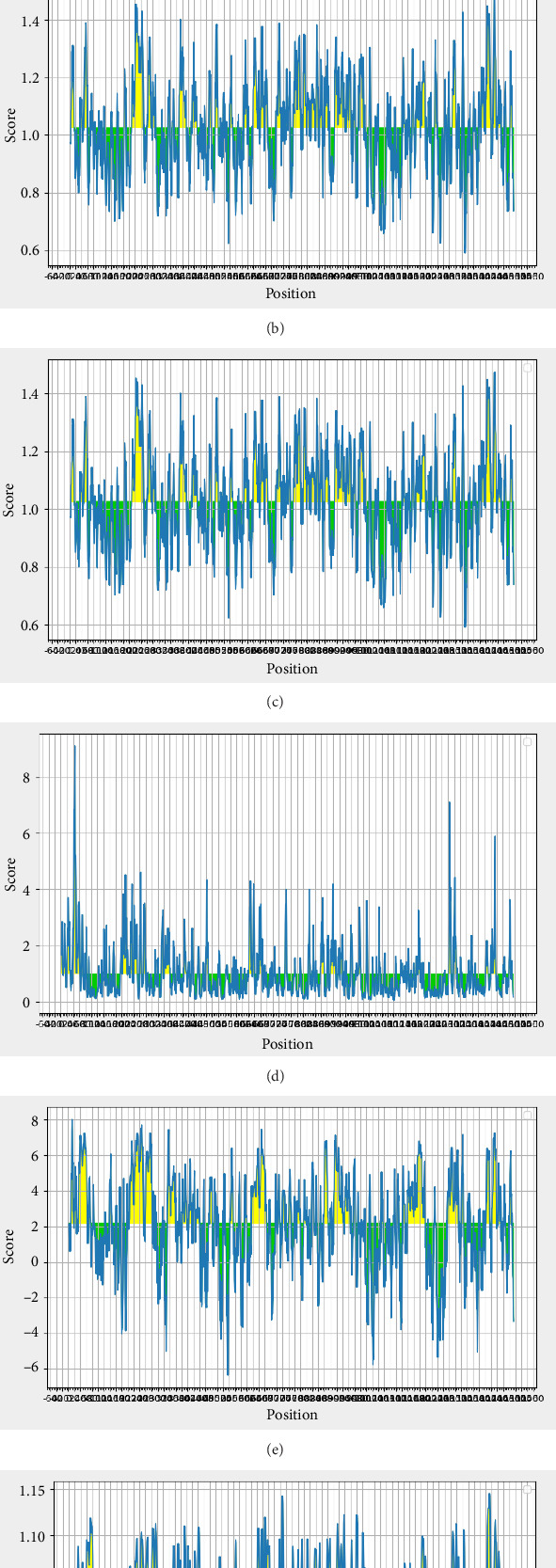
Propensity scale plots of CDPK8 protein. (a) BepiPred linear epitope prediction. (b) Antigenicity. (c) Beta-turn. (d) Surface accessibility. (e) Hydrophilicity. (f) Flexibility. On the graphs, the *Y*-axis depicts for each residue the correspondent score (averaged in the specified window), while the *X*-axis depicts the residue positions in the sequence. The tables provide values of calculated scores for each residue. The larger score for the residues might be interpreted as that the residue might have a higher probability of being part of epitope (those residues are colored in yellow on the graphs). Green color (below the threshold) indicates the unfavorable regions related to the properties of interest.

**Figure 9:**
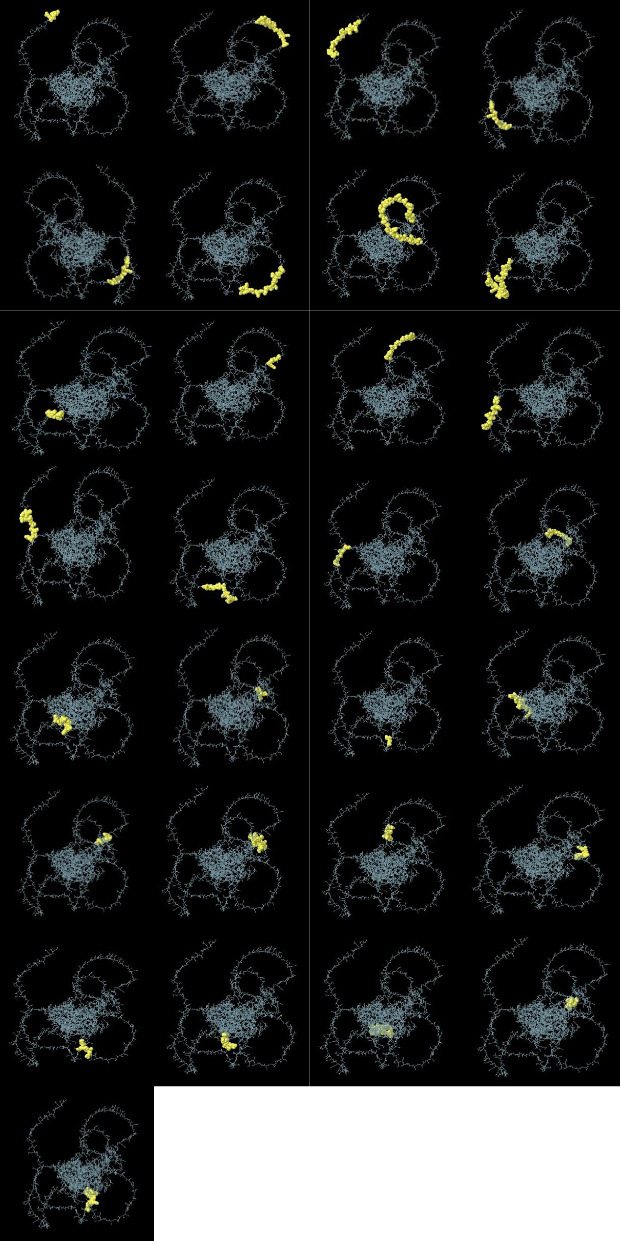
3D model of discontinuous B-cell epitopes in CDPK8 sequence. The polyprotein's bulk is represented by grey sticks, while the B-cell epitopes' discontinuous surfaces are represented by yellow surfaces.

The ABCpred tool predicted high-scoring 16-mer linear B-cell epitopes (Table 3).  Figure 7 illustrates continuous B-cell epitopes considering multiple physicochemical characteristics, assessed through the ProtScale tools. Additionally, 35 predicted epitopes with high scores were generated by the BCPreds tool, where a higher threshold score indicates increased binding affinity and specificity (Table 4). Moreover, SVM results are depicted in Table 5.  Table 6 shows the BcePred results, highlighting crucial potential epitopes that substantially contribute to defining the antigenic characteristics of the CDPK8 protein. These forecasted B-cell epitopes are categorized considering scores from a trained recurrent neural network, where a higher score shows a higher likelihood of epitope candidacy. The IEDB tool determined the average scores for beta-turn, antigenicity, BepiPred linear epitope anticipation, hydrophilicity, surface availability, and flexibility of the CDPK8 protein, yielding values of 1.025, 1.026, 2.176, 0.460, 1.012, and 1.000, respectively (Figure 8). Additionally, ElliPro analysis identified 29 conformational B-cell epitopes (Table 7), with selection based on score values ranging from 0.509 to 0.985 (Figure 9).

### 3.7. MHC-Binding Immunogenic Sequences

For the two MHC-I(10-mer) and MHC-II(15-mer) epitopes, the IC_50_ value assessment method has been used to evaluate peptide binding to mouse alleles. Additionally, Tables 8 and 9 provide a detailed analysis of immunogenicity, antigenicity, IL-4, and IFN-γ.

### 3.8. CTL Epitope Anticipation

The CTLpred tool identified CTL-specific epitopes, resulting in the prediction of 10 highly ranked 9-mer CTL epitopes within the CDPK8 protein (Table 10).

### 3.9. Allergenic and Antigenic Profiles and Solubility Evaluation

The CDPK8 antigenic profile was forecasted using the VaxiJen tool, yielding a score of 0.6227 (threshold: 0.5). AllergenFP evaluation classified the CDPK8 protein as likely nonallergenic. Furthermore, the solubility of the CDPK8 protein postexpression in *E. coli* was assessed by the SOLpro tool to be 0.968224.

### 3.10. Pattern of Simulated Immune Responses

**Figure 10:**
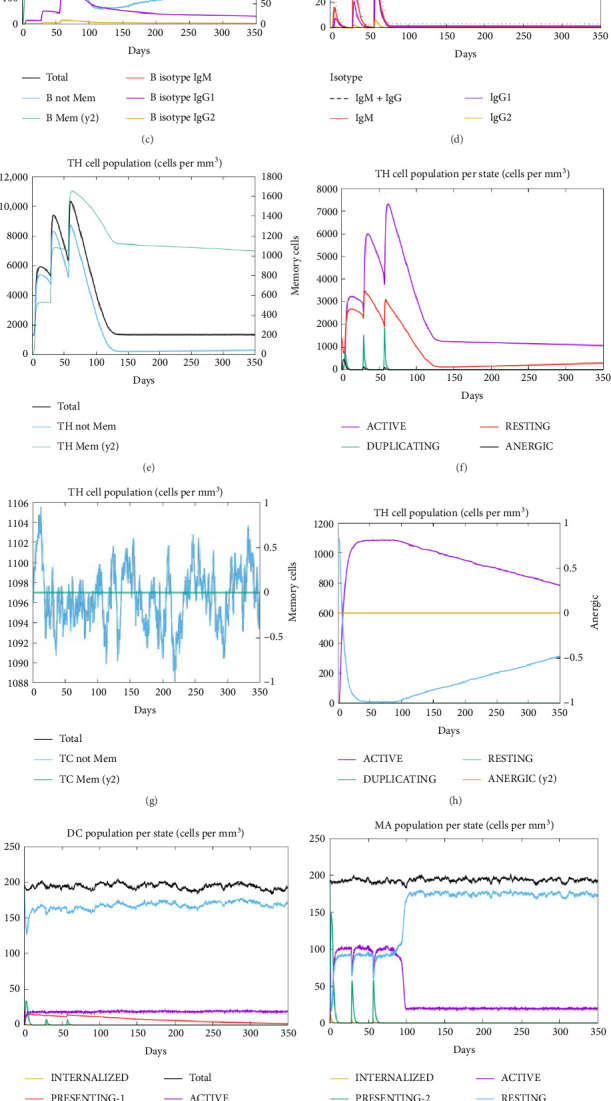
In silico immune simulation. (a) Immunoglobulin production in response to CDPK8; (b, c) B lymphocytes population; (d) plasma B lymphocytes count subdivided per isotype (IgM, IgG1, and IgG2); (e, f) TH cell (CD4+) population; (g, h) TC cell (CD8+) population; (i) dendritic cells per state; (j) macrophage population per state; (k) NK cell population; and (l) level of cytokines production (ng/mL) by CDPK8.

In all three administrations, particularly the third one, of the CDPK8 protein, an adequate titer of IgG1(∼30,000), IgM(∼40,000), and their combination (IgG + IgM) is expected (∼110,000). Based on the results obtained from C-ImmSim, a good humoral response can be considered for this vaccine (Figure 10(a)). A 2,000,000 ng/mL increase in IFN-γ, a Th-associated cytokine, occurs due to the CDPK8 protein (Figure 10(l)). The CD_4_+ T cells were present roughly 350 days after activation, which occurs 5 days after exposure to the CDPK8 vaccine and the doubling of Th cells (Figures 10(e) and 10(f)). A sustained increase in activity for several weeks following exposure was shown by T-CD_8_^+^ (T-cytotoxic) cells, with a comparable pattern observed (Figures 10(g) and 10(h)). Following antigen exposure, the overall number of natural killer (NK) cells rose for about 10 days, triggering the secretion of IFN-γ and the destruction of tachyzoite-infected cells (Figures 10(k), 10(l)). Further details are provided in Figures 10(a), 10(b), 10(c), 10(d), 10(e), 10(f), 10(g), 10(h), 10(i), 10(j), 10(k), 10(l).

## 4. Discussion

It has been more than a century since the discovery of *T. gondii*, a widely distributed zoonotic protozoan of particular concern for individuals with compromised immune systems and during pregnancy [61, 62]. In individuals with a weakened immune system, tachyzoites can invade almost all host cells with nuclei, resulting in clinical illness. However, parasites can remain as bradyzoite forms within tissue cysts in hosts, increasing the opportunistic infection risk when immune reactions are suppressed [61]. Therefore, for preventing both acute and chronic infections, it is strongly advised to implement immunoprophylactic strategies. Identifying and comprehending the precise immune-related mechanisms is essential for effectively addressing *Toxoplasma* [63, 64]. Despite significant advancements in vaccination research over the years, the development and implementation of a viable vaccine candidate for *T. gondii* have proven challenging [65–67]. The efficacy of live immunization against *T. gondii* is shown with the creation of “Toxovax,” which effectively shields sheep from congenital infection. However, these live vaccines are deemed unsafe for use in humans [68]. Regarding human vaccine advancement, the emphasis has primarily been on vector-oriented, DNA, and protein vaccines, each presenting pros and cons [65]. The progress in computer science has allowed for the virtual discovery of potential epitopes in particular sequences, resulting in decreased experimental expenses and aiding in the development of top-notch vaccine blueprints. In *T. gondii*, a set of calcium-associated molecular activities is coordinated by a certain group of protein kinases named CDPKs. Because CDPKs are not expressed in mammalian and fungal cells, they offer a promising path for additional investigation as a potential focus for toxoplasmosis immunization [69]. This study explored different aspects of the CDPK8 protein using a range of bioinformatics tools to develop a potent vaccine candidate against *T. gondii*.

Through the analysis conducted via the ProtParam tool, a range of physicochemical characteristics of the CDPK8 protein were revealed. The CDPK8 peptide sequence consists of 1498 residues, with a MW of 16.142110 kDa, suggesting its strong potential as an antigen (antigens with molecular weights ranging from 5 to 10 kDa are generally regarded as weak immunogens). This analysis was performed using artificial intelligence-based tools to ensure precise and advanced findings [70]. Additionally, using the II, the protein's stability was assessed. The findings indicated that the CDPK8 protein had an II of 62.89, categorizing it as unstable (a value exceeding 40 signifies protein instability). Here, the AI score for the CDPK8 sequence is 73.20, while its GRAVY score is −0.471. An elevated AI value indicates greater structural stability of the target protein across diverse temperature conditions, highlighting its resilience under varying thermal environments, indicating that CDPK8 is thermostable. Furthermore, the negative GRAVY value suggests a hydrophilic nature of the protein, facilitating better binding to surrounding water molecules. PTMs play a key role in controlling various cellular processes and are widely recognized for their regulatory importance [71]. Consequently, a bioinformatics analysis was conducted to investigate potential PTMs in the submitted CDPK8 protein. A considerable number of PTMs were identified within the protein sequence, including 13 N-glycosylation sites, 132 O-glycosylation sites, 198 phosphorylation sites, and 16 acetylation sites. Notably, this protein lacks transmembrane domains and is accessible to antigen-presenting cells, facilitating T- and B-cell activation for rapid immune reactions.

Assessing the protein's secondary structure plays a role in predicting its tertiary structure, involving specific constraints like beta-turns or alpha helices. The secondary structure of a polypeptide chain is largely shaped by the specific arrangement of hydrogen bonds formed between carboxyl oxygen and amino hydrogen atoms, with *β*-structures and *α* -helices being the most common configurations [72]. The results obtained revealed that the CDPK8 protein had 31.38% alpha-helix, 58.54% random coil, and 10.08% extended strand elements. The alpha-helices and beta-turns within the protein's interior, characterized by strong hydrogen bond energy, play a crucial role in preserving the protein's structural integrity while facilitating strong interactions with antibodies [73]. The biological functions of proteins primarily hinge on their spatial configurations.

Predicting the tertiary structure represents the final aim in determining a protein's structure. Understanding protein structures is of utmost importance as it elucidates the relationship between structure and function [24, 32, 74]. In this study, the SWISSMODEL platform, an interactive web tool for protein structure modeling, was utilized to construct the CDPK8 protein tertiary structure. Validating the structure is a vital component of protein structure prediction, and the Ramachandran plot is a valuable tool for assessing the accuracy of experimental structures and forecasting the biological functions of proteins [75]. In order to confirm the developed 3D model, the Ramachandran plot was produced through the SWISS-MODEL method. An essential element in structural biology is recognizing was refined differences between experimental and theoretical protein structure models. To tackle this issue, the best model from the SWISS-MODEL was selected and the model further by the GalaxyRefine tool. The refined model, with a sequence identity of 63.03, underwent additional optimization by the GalaxyRefine tool. Subsequently, SWISSMODEL tool was employed to validate the first refined model, assessing various quality metrics such as Rama favored (96.3), GDT-HA (0.8631), MolProbity (1.504), RMSD (0.733), clash score (4.8), and poor rotamers (0.3). Subsequently, both the original and refined models underwent reassessment using the same methodology. Evaluating the Ramachandran plot postrefinement indicated the production of a high-quality 3D model. The CDPK8 protein exhibited promising modeling characteristics, with a substantial percentage (96.25%) of residues located within favored areas and a minimal percentage (0.72%) in the outlier region. Additionally, the SCRATCH protein tool was employed to identify 15 potential disulfide linkages across the CDPK8 sequence. Disulfide bonds are crucial for determining the structure and functionality of proteins [76]. Accurate prediction of disulfide bonds is essential for improving the 3D structure and folding processes, as they can reduce the various conformational possibilities [70].

An epitope is a small, specific part of an antigen that is recognized by the immune system, particularly by antibodies, B-cells, or T-cells. Exploring epitopes is vital for assessing protein specificity and antigenicity, aiding in comprehending the antigen functions and structures in question [24, 32]. Therefore, a peptide exhibiting appropriate characteristics can effectively engage with antibodies and typically function as an epitope. Consequently, in order to confer protection against *T. gondii*, it is essential to consider selecting T-cell epitopes in addition to B-cell epitopes. Thus, we aimed to identify immunodominant epitopes in the CDPK8 protein sequences capable of eliciting cellular and humoral immune reactions.

Infection by *T. gondii* initiates a strong immune response involving both humoral and cell-mediated components [77]. The cellular immune response is pivotal in managing acute and chronic *T. gondii* infections. T-cells, encompassing different forms of T-lymphocytes such as CD_4_^+^ and CD_8_^+^ T-cells, in conjunction with cytokines such as TNF-α, IL-2, and IFN-γ, are essential for protective immunity. In addition, cytokines like IL-10, IL-4, and IL-5 are essential in modulating immune responses, exerting significant regulatory effects [78, 79]. On the other hand, MHC molecules have a vital impact on presenting T-cell epitopes to T-cells. The peptide binding to MHC is a significant step in antigen presentation to T-cells and is a key determinant in selecting potential epitopes [80]. We utilized the IEDB tool to assess the IC_50_ values of peptides interacting with MHC-I and MHC-II molecules concerning CDPK8. Based on the IEDB results, both MHC-I and MHC-II molecules demonstrated a strong interaction with the T-cell epitopes on CDPK8. Lower percentile ranks (or IC_50_ values) indicate a higher affinity level, suggesting a more effective T-cell epitope, or conversely. A significant hurdle in subunit vaccine development lies in identifying peptides capable of activating CTL. To address this, we utilized the CTLpred tool to predict CTL epitopes and selected the top 10 epitopes for the CDPK8 protein based on its predictions. CTLpred stands out as a specialized tool for predicting CTL epitopes, playing a vital role in vaccine development. This method relies on sophisticated ML techniques such as SVMs and ANNs. By utilizing consensus and combined prediction methods, the CTLpred tool enhances the accuracy of epitope prediction. Compared to individual techniques, such as SVM and ANN, combined and consensus prediction approaches offer greater specificity and sensitivity [56].

Antibodies produced by B-cells, along with IFN-γ, play a significant role in the defense against *Toxoplasma gondii* and contribute to long-term immunity. [81, 82]. Moreover, these antibodies aid immune cells such as macrophages (MQs) in efficiently clearing *T. gondii* and preventing infection recurrence [83]. We employed various methods and online tools to anticipate B-cell epitopes, enhancing the precision and reliability of our predictions. The examination of linear B-cell epitopes identified promising regions within the CDPK8 protein, characterized by optimal index values. These findings were obtained using several online tools, including BCPREDS and BcePred, alongside SVM. Additionally, IEDB, ABCpred, and Ellio pro were also utilized in this evaluation. For instance, our findings indicated the prediction of 35 potential antigen epitopes in CDPK8 using BCPREDS. Furthermore, through SVM analysis, we identified and listed 10 epitopes in a table based on our recommendations. Moreover, the anticipation accuracy of BcePred for models utilizing different characteristics ranges from 52.92% to 57.53%, allowing for the identification of B-cell epitopes by leveraging diverse physicochemical characteristics such as flexibility/mobility, accessibility, hydrophilicity, exposed surface, polarity, and turns [84]. Additionally, we have introduced a BcePred tool post our evaluation, aimed at forecasting linear B-cell epitopes within a protein sequence. The predictive accuracy of BcePred across models incorporating diverse properties varies between 52.92% and 57.53%. This platform also allows individuals to anticipate B-cell epitopes by taking into account turns, polarity, hydrophilicity, surface exposure, availability, and mobility/flexibility [45]. Moreover, the ABCpred database utilizes an ANN to forecast B-cell epitopes located in antigen sequences. This tool can achieve a precision of 65.93% in epitope prediction through a recurrent neural network [43].

Presently, the identification of allergenic proteins holds significant importance because of the use of altered proteins in food production, biopharmaceuticals, therapeutic approaches, and more [58]. The capability to anticipate allergenic properties is crucial to ensure that vaccine candidates exhibit low allergenicity. The results from antigenicity analysis (conducted using the VaxiJen tool) and allergenicity assessment (performed via the AlgPredFP tool) showed that the CDPK8 protein is immunogenic yet nonallergenic.

In the immune simulations, actual immune responses to peptide antigens were reported, with a consistent increase in responses observed each time there was repeated exposure to CDPK8. The findings clearly demonstrate the persistence of memory B-cells for several months and a notable stimulation of helper T-cells, indicating their maturation. Particularly noteworthy was the sustained elevation of IFN-γ and IL-2 levels following the third dose, indicating a substantial presence of helper T-cells, thereby bolstering a robust humoral immune response [77, 85]. Also, similar to Masoud Foroutan et al. [86], Ali Taghipour et al. [87], and Ali Dalir Ghaffari et al. [88] studies, which were conducted on CDPKs proteins, our study indicated CDPK8 exhibited high immunogenicity scores, and using in silico techniques, a number of potential CDPK protein epitopes were found, suggesting that these techniques have potential applications in immunogenic epitope prediction.

To confirm the immunogenicity of the anticipated sequences, validation in an appropriate mouse model using various bioinformatics tools is recommended. Therefore, it is strongly advised to undertake further studies utilizing in virtual simulations and in biological systems approaches to assess the protein's efficacy as a potential vaccine target.

## 5. Conclusion

We examined the physicochemical attributes, potential B- and T-cell epitopes, tertiary and secondary structures, transmembrane domains, cellular subcompartmentalization, and other characteristics of the CDPK8 protein. CDPK8 exhibited notable surface accessibility, flexibility, antigenicity, and hydrophilicity indices. Epitope prediction results from diverse bioinformatics databases revealed the CDPK8 protein contains highly effective B- and T-cell epitopes, highlighting its potential as a crucial element in a *T. gondii* vaccine. Our findings indicate that utilizing in silico tools for structural and functional predictions of the CDPK8 protein can help mitigate risks of failure in laboratory studies. This investigation lays a crucial foundation for future research endeavors and the design of a successful vaccine for chronic and acute toxoplasmosis through various strategies.

## Figures and Tables

**Table 1:** Acetylation sites predicted using GPS-PAIL 2.0 server with high threshold.

ID	Position	Peptide	HAT	Score	Cutoff
1	46	SGREDEQKRRRGEDS	EP300	0.486	0.42
2	46	SGREDEQKRRRGEDS	KAT2B	1.697	1.343
3	176	FETFEDHKYIYLVLE	KAT8	8.8	7.222
4	263	AIGAGREKEGRSGGA	KAT2B	1.532	1.343
5	272	GRSGGAGKDGQEEER	EP300	0.496	0.42
6	272	GRSGGAGKDGQEEER	KAT8	11.6	7.222
7	363	REAKSSLKLAESVLQ	KAT2A	1.391	1.382
8	637	RSAATDSKQPESAVT	CREBBP	2.004	1.348
9	656	EGDGATEKGERTPGK	EP300	0.903	0.42
10	663	KGERTPGKKGWEPGV	KAT2B	1.541	1.343
11	664	GERTPGKKGWEPGVL	KAT2B	1.358	1.343
12	1148	SDASSVAKRAEHCSA	CREBBP	1.452	1.348
13	1301	WGASGNSKKLRREGE	KAT2A	1.754	1.382
14	1301	WGASGNSKKLRREGE	KAT8	7.4	7.222
15	1302	GASGNSKKLRREGEA	KAT2A	2.13	1.382
16	1436	DSGSPDQKRDLLGEG	KAT2B	2.055	1.343

**Table 2:** Disulfide bonds predicted using scratch pro.

Bond index	Cys1_position	Cys2_position
1	139	1455
2	935	950
3	568	576
4	732	765
5	88	1275
6	97	1231
7	239	292
8	807	814
9	119	1207
10	106	1172
11	998	1020
12	185	1153
13	1096	1099
14	592	600
15	157	1104

**Table 3:** Predicted B-cell epitope using ABCpred server.

Rank	Sequence	Start position	Score	VaxiJen score	Allergenicity	Water solubility
1	STESGDRGDRRAGSTE	892	0.94	1.5534	NON-ALLERGEN	Good
1	HVTPDNMSLYRAVLKQ	1462	0.94	−0.0863	NON-ALLERGEN	Good
1	SFEAADASEWNEGCAE	1159	0.94	−0.3599	ALLERGEN	Good
2	DRRAGSTERGDAGLKT	900	0.92	1.0923	NON-ALLERGEN	Good
2	QDARFARTRQRAGQQP	198	0.92	0.9399	ALLERGEN	Good
2	AFLVVEPPPFLAFLSS	1248	0.92	1.1482	ALLERGEN	Poor
3	GRRETSPSAYIRGASP	818	0.91	0.6587	NON-ALLERGEN	Good
3	ACGAWDCLDAPRLCVP	801	0.91	0.0492	ALLERGEN	Poor
3	QRAGQQPDDEARPAGP	207	0.91	1.2815	ALLERGEN	Good
3	QWAQAELCDGDAGAVQ	1268	0.91	0.2223	ALLERGEN	Good
3	HRGVLAPDDIVWGLNV	1072	0.91	1.0009	NON-ALLERGEN	Poor
4	SEWNEGCAENDEGANE	1166	0.90	0.3747	ALLERGEN	Good
5	KKGWEPGVLRRMDAAT	663	0.89	−0.9373	NON-ALLERGEN	Good
5	GATEKGERTPGKKGWE	652	0.89	1.0093	ALLERGEN	Good
5	PSSETAPGLGVDERQL	420	0.89	0.7269	NON-ALLERGEN	Good
5	GGGRDSGDRDRGDRCR	225	0.89	1.2654	ALLERGEN	Good
6	FRSSKATDDEGRRGQP	856	0.88	1.3679	NON-ALLERGEN	Good
6	CSSWHVAPPGTKKRAE	732	0.88	−0.0398	NON-ALLERGEN	Good
6	DSSEGDGATEKGERTP	646	0.88	1.3179	ALLERGEN	Good
6	SGSRDSSTKRQAVAQL	53	0.88	0.6047	NON-ALLERGEN	Good
6	PAGPRDGGGRDSGDRD	219	0.88	0.7824	ALLERGEN	Good
6	DRDKDGYITYDEFLLL	1482	0.88	1.4294	ALLERGEN	Good
6	PDMRAASPLRNAFESD	1127	0.88	1.1405	NON-ALLERGEN	Good
7	PSSPLHPSRPRWGAEV	980	0.87	1.2950	NON-ALLERGEN	Good
7	TKKRAEAVRPFASLSP	742	0.87	0.4940	NON-ALLERGEN	Good
7	GVHGERGSVARNFSCS	718	0.87	0.8027	NON-ALLERGEN	Good
7	ADSWTIHDVVARMDTD	1309	0.87	−0.4316	ALLERGEN	Good
8	PSVPSACVEGFLSRTS	944	0.86	0.5862	ALLERGEN	Poor
8	GLGVDERQLPEASADL	427	0.86	0.4240	NON-ALLERGEN	Good
8	DLAQETRAHPGERSTA	340	0.86	0.6851	NON-ALLERGEN	Good
8	ESLREASDDSGSPDQK	1421	0.86	0.8935	ALLERGEN	Good
8	CGEEEAPLEVHQFLPL	1104	0.86	0.5822	ALLERGEN	Good
8	DGDEDSHVSLEKREAP	11	0.86	0.7820	NON-ALLERGEN	Good
9	SAYIRGASPSNPRLLP	825	0.85	1.0832	NON-ALLERGEN	Poor
9	SSTSLSPSPSSASLAS	766	0.85	0.5339	ALLERGEN	Poor
9	AFHRGLHGVHGERGSV	711	0.85	0.1724	NON-ALLERGEN	Good
9	SGREDEQKRRRGEDSG	39	0.85	1.6100	ALLERGEN	Good
9	SVLQSPPSTSPFGLSY	367	0.85	0.1994	ALLERGEN	Poor

**Table 4:** BCPred predictions of linear B-cell epitopes from CDPK8 protein.

No.	Position	Epitope	Score	VaxiJen score	Allergenicity	Water solubility
1	203	ARTRQRAGQQPDDEARPAGP	1	1.3372	ALLERGEN	Good
2	259	AGREKEGRSGGAGKDGQEEE	1	2.4611	NON-ALLERGEN	Good
3	654	TEKGERTPGKKGWEPGVLRR	1	0.2254	NON-ALLERGEN	Good
4	1167	EWNEGCAENDEGANEGEERG	1	0.5012	ALLERGEN	Good
5	1274	LCDGDAGAVQRQEKEGERGG	1	1.0780	ALLERGEN	Good
6	1279	AGAVQRQEKEGERGGWGASG	1	1.6654	NON-ALLERGEN	Good
7	1397	LSHGIMSSSDSSPQSGNSSG	1	1.1532	ALLERGEN	Good
8	39	SGREDEQKRRRGEDSGSRDS	0.999	1.4922	ALLERGEN	Good
9	202	FARTRQRAGQQPDDEARPAG	0.999	1.1114	ALLERGEN	Good
10	226	GGRDSGDRDRGDRCRGDRDN	0.999	1.1871	ALLERGEN	Good
11	231	GDRDRGDRCRGDRDNGDGEE	0.999	1.3932	ALLERGEN	Good
12	234	DRGDRCRGDRDNGDGEERHE	0.999	1.3222	ALLERGEN	Good
13	258	GAGREKEGRSGGAGKDGQEE	0.999	2.2989	NON-ALLERGEN	Good
14	266	RSGGAGKDGQEEERGNLDSL	0.999	1.6179	NON-ALLERGEN	Good
15	646	DSSEGDGATEKGERTPGKKG	0.999	1.5173	ALLERGEN	Good
16	653	ATEKGERTPGKKGWEPGVLR	0.999	0.5099	NON-ALLERGEN	Good
17	854	AAFRSSKATDDEGRRGQPGA	0.999	1.2269	ALLERGEN	Good
18	892	STESGDRGDRRAGSTERGDA	0.999	1.4532	NON-ALLERGEN	Good
19	1165	ASEWNEGCAENDEGANEGEE	0.999	0.6714	ALLERGEN	Good
20	1273	ELCDGDAGAVQRQEKEGERG	0.999	0.7385	ALLERGEN	Good
21	1396	SLSHGIMSSSDSSPQSGNSS	0.999	0.9454	ALLERGEN	Good
22	38	ESGREDEQKRRRGEDSGSRD	0.998	1.3323	ALLERGEN	Good
23	201	RFARTRQRAGQQPDDEARPA	0.998	1.0882	ALLERGEN	Good
24	227	GRDSGDRDRGDRCRGDRDNG	0.998	1.1752	ALLERGEN	Good
25	232	DRDRGDRCRGDRDNGDGEER	0.998	1.2367	ALLERGEN	Good
26	257	IGAGREKEGRSGGAGKDGQE	0.998	2.1739	ALLERGEN	Good
27	618	SAASPPSQRPSPRSAATDSK	0.998	0.9893	ALLERGEN	Good
28	644	TCDSSEGDGATEKGERTPGK	0.998	1.4792	ALLERGEN	Good
29	649	EGDGATEKGERTPGKKGWEP	0.998	1.0651	ALLERGEN	Good
30	811	PRLCVPGGRRETSPSAYIRG	0.998	0.5180	NON-ALLERGEN	Good
31	815	VPGGRRETSPSAYIRGASPS	0.998	0.7479	NON-ALLERGEN	Good
32	855	AFRSSKATDDEGRRGQPGAI	0.998	1.3579	NON-ALLERGEN	Good
33	891	QSTESGDRGDRRAGSTERGD	0.998	1.5435	NON-ALLERGEN	Good
34	926	PPREVTRDPCSTVASSTSPS	0.998	0.5098	ALLERGEN	Good
35	1272	AELCDGDAGAVQRQEKEGER	0.998	0.6501	ALLERGEN	Good

**Table 5:** B-cell linear epitopes predicted using SVMTriP server.

Rank	Location	Epitope	Score	VaxiJen score	Allergenicity
1	1362–1377	FDGLVSYRDLLRLLSL	1.000	−0.0545	ALLERGEN
2	1217–1232	SLRSLLHLAALTSSCA	0.955	0.1877	ALLERGEN
3	455–470	KFSDSIILADFGLARR	0.741	−0.3056	ALLERGEN
4	302–317	TQLLSALSYLHRKGIA	0.697	−0.0304	NON-ALLERGEN
5	1088–1103	ARELVTSECESCLVKV	0.619	0.6286	ALLERGEN
6	1016–1031	AIFHCRFLLLLGATSW	0.617	1.3770	NON-ALLERGEN
7	1056–1071	LEVRVLREVFSVLDRG	0.536	−0.2993	ALLERGEN
8	550–565	EAEVSSKILTTHIAFK	0.454	0.8482	ALLERGEN
9	1135–1150	LRNAFESDASSVAKRA	0.454	0.3528	ALLERGEN
10	156–171	LCRQLDHPNIARLFET	0.440	−0.7240	ALLERGEN

**Table 6:** The predicted B-cell epitopes, using physico-chemical properties based on BcePred server.

Prediction parameter	Epitope sequence
Hydrophilicity	DLPQDGDEDSHVS, EKREAPEPER,ESASAESGREDEQKRRRGEDSGSRDSSTKRQA, RVCKDENHAE, EYCRGGD,RTRQRAGQQPDDEARPAGPRDGGGRDSGDRDRGDRCRGDRDNGDGEERHEGV, GAGREKEGRSGGAGKDGQEEERGNLDS,KSRDEGEDLA, HPGERSTAREAKSS,QSPPSTS, SYGSSTR,SSPAEEG, EREGETHSR,PSSETAPG, ETKFSDS,GGAARGERATRRR, RAASSGG,YDEVQSDM, EGNSEAEVSSK,VSEECKD, NCSHGSTQ,SQRPSPRSAATDSKQPESAVTCDSSEGDGATEKGERTPGKKG, AKASSST,PRSSSSPA, GTKKRAEA,SPSPSSAS, SPSPSSAS,PGGRRETSPSA, RGASPSNP,RSSKATDDEGRRGQPG, QSTESGDRGDRRAGSTERGDAG,KTGSSPQASR, TRDPCST,ASSTSPS, SRTSSPQETSA,PPDPDQAA, AREMEDE,VTSECESC, KVCGEEEAP,SRSSASPD, ESDASSV, CSASPSS, EAADASE, NEGCAENDEGANEGEERGGREG, RGGDVGRED,CDGDAGAVQRQEKEGERGG, GASGNSKK,RREGEADS, ARMDTDGSGNIE,MSSSDSSPQSGNSSG, LREASDDSGSPDQKRD, GEGEGAPEG, QAVDRDKDGY
Flexibility	DLPQDGDE, SHVSLEK,ESASAESGREDEQKRRRGEDSGSRDSSTKR, GQLKRVRS,ARFARTRQ, RPAGPRDGGGRDSGDRDRGDRCRGDRDNGDGE,VAIGAGREKEGRSGGAGKDGQEEER, VHPFKSRDEG,TRAHPGERSTAREAK, SVLQSPPST,FGLSYGSST, DVLEREGETHSRLSSSLPS,MPETKFS, GLARRFS,GAARGERATR, RAASSGGL,RHVSEEC, CNCSHGS,EFLSAASRSAASPPSQRPSP, SAATDSKQP,SAVTCDSSEGDGATEKGERTPGK, ELWAKASSSTLSMPRSSSS,HGVHGERG, HVAPPGTKKR,ASLSPSISS, SLCSSTSLSPSPSS,VPLSPSPSS, LCVPGGRRETS,AYIRGASPSN, PHLPKSVSS,AAFRSSKATDDEGRRGQ, RALEKRA,ARLQSTESGDRGDRRAGSTER, DAGLKTGSSPQA,STVASSTSP, EGFLSRTSSPQE,SPLHPSR, SVLDRGH,ELVTSEC, PLLSRSSAS,NAFESDA, EHCSASPSS,EGANEGEERGGR, GAVQRQEKEGERGGWGASGNSKKLRREG,ARMDTDGS, DLAGRESL,HGIMSSSDSSPQSGNSS, ESLREASDDSGSPDQK,QAVDRDK
Accessibility	MERRPLDLPQDGDEDSHVSLEKREAPEPERVLES, SAESGREDEQKRRRGEDSGSRDSSTKRQAVAQLRDEDRLS,GQLKRVRSEG, LERVVKRVCKDENHAELPQVRKEIA,RQLDHPNIAR, ETFEDHKYIY,HARQDARFARTRQRAGQQPDDEARPAGPRDGGGRDSGDRDRGDRCRGDRDNGDGEERHEGV, GAGREKEGRSGGAGKDGQEEERGNLDS,LSYLHRKGIAHRDLKLEN, HPFKSRDEGEDLAQETRAHPGERSTAREAKSSLK,QSPPSTSP, SYGSSTRVYSSPAEE,LEREGETHSRLS, PSSETAP,VDERQLPEAS, SLMPETKFSDS,ARGERATRRRFLRA, KGLPYDEVQSD,PFEGNSEAEVSSK, KEPCWRHVSEECKDLIRRLLHNP,ASPPSQRPSPRSAATDSKQPESAV, GATEKGERTPGKKGWEPG,RRMDAAT, QRELWAK,SMPRSSSSPA, APPGTKKRAEAVRP,VPGGRRETSPSAY, RGASPSNPRL,PKSVSSP, FRSSKATDDEGRRGQPGA,SRALEKRAGQIARLQSTESGDRGDRRAGSTERGDA, KTGSSPQASRISPPREVTRDPCST,SRTSSPQETSA, SPRIPPSSPLHPSRPRWGAE,VPPDPDQAA, PLQRALR,AREMEDELEVR, EEEAPLE,SRSSASPD, RNAFESD,KRAEHCS, DASEWNE,ENDEGANEGEERGGREG, GAVQRQEKEGERGG,ASGNSKKLRREGEADS, RMDTDGS,FRYFDRSF, SYRDLLR,SSSDSSPQSGNSS, HPESLREASDDSGSPDQKRDLLGE,EHVTPDNMSLYRAVLK, QAVDRDKDGYITY
Turns	QLDHPNIA, FLCNCSHGST,MSSSDSSPQ
Exposed surface	—
Polarity	—
Antigenic propensity	LSVPGQL, VRSEGCVLKRQQLICSSTLPITTCYHLGEVIGVGTWCQV,SGLERVVKRVCKD, LCRQLDH,DHKYIYLVLEYCRG, DLLSWLH,VDDRPICSELQ, LENILLVHPFKS,SVLQSPPS, LSSSLPS,CLLSSLMP, RVGTLYYLSPFLLKGLPY,GVILYMLIS, VSSKILT,PCWRHVS, LIRRLLHNPFLCNCSHGSTQLCPSLVRPLS,LTHPWFV, SCSSWHV,ISSGSLCSSTSLS, SSVFPVPLSPSP,PRLCVPGGR, PRLLPHLPKSVSS,CVEGFLS, IPPSSPL,EVPGCLLR, IFHCRFLLLLG,ELEVRVLREVFSVLDR, IVWGLNV,ELVTSECESCLVKVCGEE, PLEVHQFLPLLSRS,ECPLGNV, PSLRSLLHL,PPVGYILPYLLNLH, FLVVEPPPFL,WTIHDVV, IEFVEFV,FDGLVSYRDLLRLLSLQP, ECFLTDLEHV,YDEFLLL

**Table 7:** Predicted discontinuous B-cell epitope residues of the CDPK8 sequence.

No.	Residues	Number of residues	Score
1	A:M1, A:E2, A:R3	3	0.985
2	A:D224, A:G225, A:G226, A:G227, A:R228, A:D229, A:S230, A:G231, A:D232, A:R233, A:D234, A:R235, A:G236, A:D237, A:R238, A:C239, A:R240	17	0.981
3	A:R4, A:P5, A:L6, A:D7, A:L8, A:P9, A:Q10, A:D11, A:G12, A:D13, A:E14, A:D15, A:S16, A:H17, A:V18, A:S19, A:L20	17	0.958
4	A:R626, A:P627, A:S628, A:P629, A:R630, A:S631, A:A632, A:A633, A:T634, A:D635, A:S636, A:K637	12	0.874
5	A:E408, A:G409, A:E410, A:T411, A:H412, A:S413, A:R414	7	0.845
6	A:L415, A:S416, A:S417, A:S418, A:L419, A:P420, A:S421, A:S422, A:E423, A:T424, A:A425, A:P426, A:G427, A:L428, A:G429, A:V430, A:D431, A:E432, A:R433	19	0.837
7	A:S371, A:P372, A:P373, A:S374, A:T375, A:S376, A:P377, A:F378, A:G379, A:L380, A:S381, A:Y382, A:G383, A:S384, A:S385, A:T386, A:R387, A:V388, A:Y389, A:S390, A:S391, A:P392, A:A393, A:E394, A:E395, A:G396, A:V397, A:A398, A:P399, A:A400, A:P401, A:A402, A:D403, A:V404, A:L405, A:E406	36	0.831
8	A:D43, A:E44, A:Q45, A:K46, A:R47, A:R48, A:R49, A:G50, A:E51, A:D52, A:S53, A:G54, A:S55, A:R56, A:D57, A:S58, A:S59, A:T60, A:K61, A:R62, A:Q63, A:A64, A:V65, A:A66, A:Q67, A:L68	26	0.829
9	A:D70, A:E71, A:D72, A:R73	4	0.819
10	A:R218, A:P219, A:A220, A:G221, A:P222	5	0.804
11	A:G241, A:D242, A:R243, A:D244, A:N245, A:G246, A:D247, A:G248, A:E249, A:E250, A:R251, A:H252	12	0.8
12	A:V31, A:L32, A:E33, A:S34, A:A35, A:S36, A:A37, A:E38, A:S39, A:G40, A:R41, A:E42	12	0.79
13	A:E21, A:K22, A:R23, A:E24, A:A25, A:P26, A:E27, A:P28, A:E29	9	0.787
14	A:Q638, A:P639, A:E640, A:S641, A:A642, A:V643, A:T644, A:C645, A:D646, A:S647, A:S648, A:E649, A:G650, A:D651, A:G652, A:A653, A:T654, A:E655	18	0.774
15	A:S618, A:A619, A:A620, A:S621, A:P622, A:P623, A:S624, A:Q625	8	0.746
16	A:E264, A:G265, A:R266, A:S267, A:G268, A:G269, A:A270, A:G271, A:K272, A:D273	10	0.715
17	A:L74, A:S75, A:V76, A:P77, A:G78, A:Q79, A:L80, A:K81	8	0.692
18	A:G274, A:Q275, A:E276, A:E277, A:E278	5	0.662
19	A:G657, A:E658, A:R659	3	0.628
20	A:R605, A:P606, A:L607, A:S608, A:A609, A:E610, A:F611, A:L612, A:S613, A:A614, A:A615, A:S616, A:R617	13	0.626
21	A:A209, A:G210, A:Q211, A:Q212, A:P213, A:D214, A:D215	7	0.596
22	A:S353, A:T354, A:A355, A:E357, A:A358, A:K359, A:S360, A:S361, A:L362, A:K363, A:L364, A:A365, A:E366, A:S367, A:V368, A:L369, A:Q370	17	0.591
23	A:E253, A:G254, A:V255, A:A256, A:I257, A:G258, A:A259, A:G260, A:R261	9	0.591
24	A:A347, A:H348, A:P349, A:G350, A:E351, A:R352	6	0.588
25	A:Q434, A:L435, A:P436, A:E437, A:A438, A:S439	6	0.576
26	A:V83, A:R84, A:S85, A:E86, A:G87, A:C88, A:V89, A:L90	8	0.56
27	A:L589, A:C590, A:N591, A:C592, A:S593, A:H594, A:G595, A:S596, A:T597, A:Q598, A:L599, A:C600, A:P601, A:S602, A:L603, A:V604	16	0.559
28	A:G280, A:N281, A:L282, A:D283, A:S284, A:L285, A:V286	7	0.528
29	A:T660, A:P661, A:G662, A:K663, A:K664, A:G665, A:W666, A:E667, A:P668, A:G669, A:V670, A:L671, A:R672	13	0.509

**Table 8:** Percentile rank for CDPK8 binding to MHC-I molecules.

Allele	Start–end	Peptide sequence	Percentile rank	Immunogenicity
H-2-Db	830–839	GASPSNPRLL	0.13	−0.2055
	959–968	SSPQETSAVL	0.26	−0.08345
	1414–1423	SSGFMHPESL	0.32	−0.0333

H-2-Kb	1489–1498	ITYDEFLLLM	0.25	0.20634
	1366–1375	VSYRDLLRLL	0.34	0.08844
	707–716	SSSPAFHRGL	0.36	0.18117

H-2-Kk	548–557	NSEAEVSSKI	0.15	−0.21178
	1107–1116	EEAPLEVHQF	0.22	0.08672
	145–154	AELPQVRKEI	0.23	−0.16214

H-2-Dd	959–968	SSPQETSAVL	0.1	−0.08345
	1212–1221	VALPPSLRSL	0.1	−0.25008
	770–779	LSPSPSSASL	0.13	−0.56134

H-2-Kd	1356–1365	RYFDRSFDGL	0.14	0.09834
	504–516	TLYYLSPFLL	0.32	−0.09494
	1237–1246	GYILPYLLNL	0.45	−0.00594

H-2-Ld	1240–1249	LPYLLNLHAF	0.03	0.01026
	975–984	SPRIPPSSPL	0.1	−0.17271
	960–969	SPQETSAVLL	0.1	0.00504

*Note:* Low percentile rank = good binders; IC_50_ values = percentile rank.

**Table 9:** Percentile rank for CDPK8 binding to MHC-II molecules, antigenicity, IFN-γ <, and IL-4 induction.

Allele	HTL epitope	Percentile rank	Antigenicity	IFN-γ inducing		IL-4 inducing	
				Result	Score	Result	SVM score
H2-IAD	SPAEEGVAPAPADVL	0.02	0.41842	Positive	0.63154701	Positive	0.63
H2-IAD	YSSPAEEGVAPAPAD	0.05	0.31907	Positive	0.2098475	Positive	0.37
H2-IAD	PAEEGVAPAPADVLE	0.13	0.34839	Positive	0.92649865	Positive	0.83
H2-IAB	SAAFRSSKATDDEGR	0.38	−0.12862	Negative	−0.066055374	Negative	−0.13
H2-IAB	LSAAFRSSKATDDEG	0.59	−0.14173	Negative	−0.42009181	Negative	−0.24
H2-IAB	SSGFMHPESLREASD	0.63	−0.01489	Negative	−0.84895376	Negative	−0.33
H2-IED	EADSWTIHDVVARMD	0.63	0.33524	Positive	0.19144298	Positive	0.25
H2-IED	QPGAISVSRALEKRA	0.91	−0.08184	Positive	1.0455878	Positive	0.76
H2-IED	DARFARTRQRAGQQP	0.92	0.15346	Positive	0.24482918	Positive	0.51

**Table 10:** Predicted CDPK8 epitopes by CTLpred.

Peptide rank	Start position	Sequence	Score (ANN/SVM)
1	*123*	TAVERFSGL	0.97/1.2247748
2	1042	RALRTVVAR	0.85/1.3389902
3	483	TRRRFLRAA	0.93/1.2064492
4	1367	SYRDLLRLL	0.48/1.5713106
5	130	GLERVVKRV	0.99/0.95014682
6	736	HVAPPGTKK	0.66/1.2238325
7	1232	AGPPVGYIL	0.97/0.91185455
8	482	ATRRRFLRA	0.78/1.0996763
9	503	TRVGTLYYL	0.18/1.6835364
10	1059	RVLREVFSV	0.51/1.2320759

## Data Availability

The data that support the findings of this study are available from the corresponding author upon reasonable request.

## Supporting Information

Links to all bioinformatics web servers used in this study are provided in Supporting Table 1.
